# Real-time multisite invasive neural recording during downhill skiing in Parkinson’s disease: a case report

**DOI:** 10.3389/fnins.2025.1564058

**Published:** 2025-04-14

**Authors:** Rodrigo Fernández-Gajardo, Ro’ee Gilron, Amelia Hahn, Philip A. Starr

**Affiliations:** ^1^Department of Neurological Surgery, University of California, San Francisco, San Francisco, CA, United States; ^2^Faculty of Medicine, Department of Neurological Sciences, University of Chile, Santiago, Chile

**Keywords:** brain-sensing devices, Parkinson’s disease, oscillatory dynamics, downhill skiing, wireless neural interfaces

## Abstract

**Introduction:**

Invasive recording of neural activity provides valuable insights into Parkinson’s disease (PD). Bidirectional sensing devices enable wireless neural data collection during everyday activities, but neural signals during complex outdoor sports remain unexplored.

**Methods:**

We recorded neural data from a 57-year-old PD patient using bilateral implanted pulse generators connected to subthalamic nucleus (STN) and motor cortex leads. Recordings were performed in two settings: in-clinic during a computer-controlled task and outdoors during downhill skiing. Neural data were analyzed for power spectral density (PSD) and coherence across different frequencies.

**Results:**

In-clinic recordings demonstrated movement-related cortical and STN beta desynchronization with cortical gamma increase. Skiing similarly induced STN beta desynchronization but also elicited low-gamma activity (30–60 Hz) and unique finely-tuned gamma (FTG) activity at 85 Hz in the off-medication state, predominantly in the less affected hemisphere. Tremor-related cortical beta suppression was observed during stopping, with prominent 10 Hz activity associated with resting tremor.

**Conclusion:**

Real-time multisite neural recordings during a complex outdoor activity revealed distinct neural signatures compared to in-clinic tasks. The findings suggest that self-cued, learned motor tasks elicit unique frequency bands and highlight differences based on disease asymmetry and medication state.

## 1 Introduction

Invasive recording of field potentials has provided insight into the neural mechanisms underlying PD motor signs. The recent advent of bidirectional sensing devices that can wirelessly stream neural data has allowed the study of neural signals during everyday activities at home (Gilron et al., 2021). However, study of neural data during highly learned outdoor sports activities, such as downhill skiing, have not been explored. Neural signals during sports demanding precise coordination, balance, and focused attention may differ from those obtained in the home or laboratory.

## 2 Materials and methods

**Figure 1:**
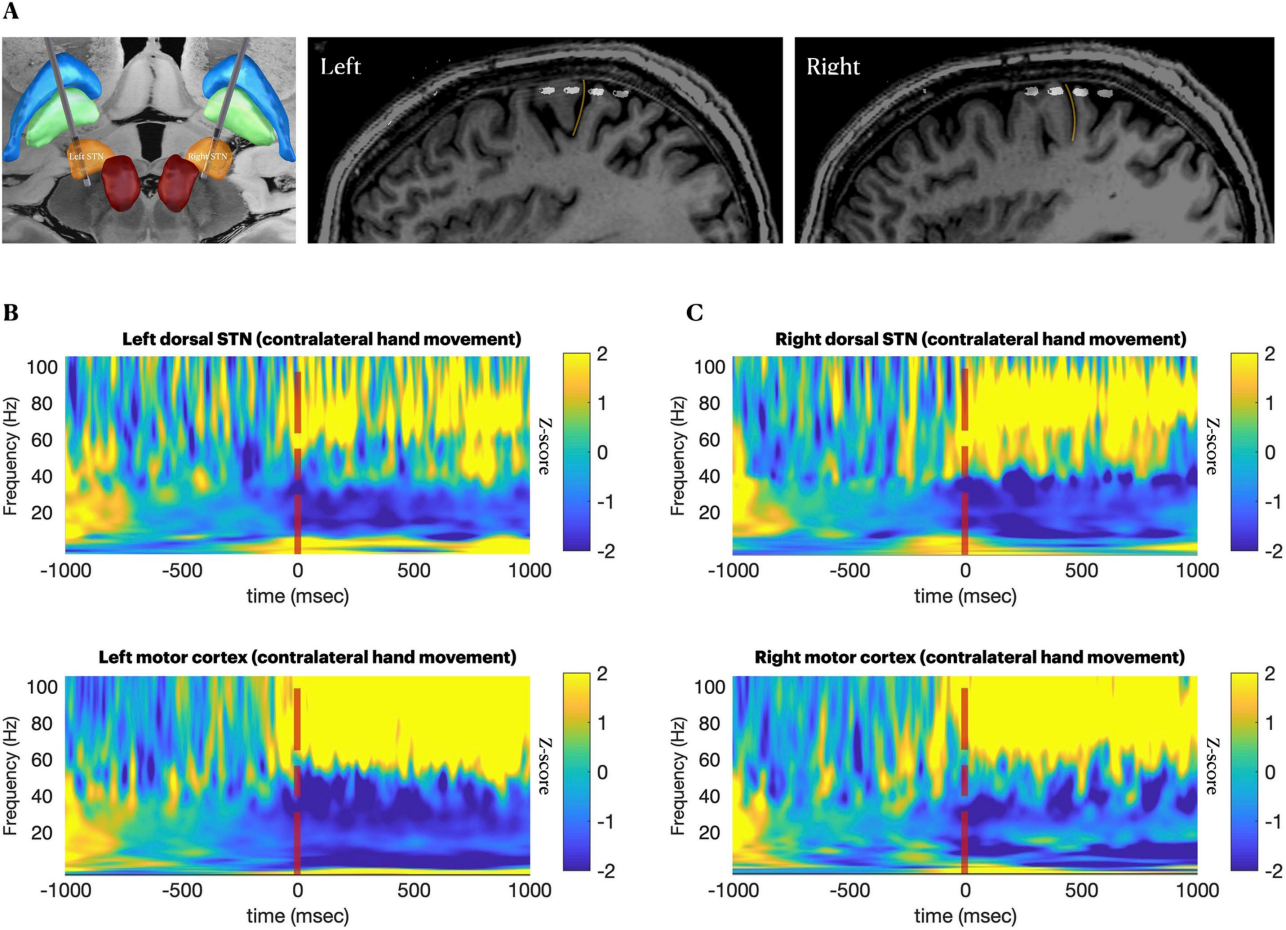
Anatomical and neurophysiological confirmation of lead positions. **(A–C)**, Locations of subthalamic and cortical leads from fusion of postoperative CT and preoperative MRI. (A), 3D visualization of subthalamic leads. **(B,C)** Perisagittal views of the subdural electrodes over sensory-motor cortices. Yellow line marks the central sulcus. **(D,E)**, spectrograms providing neurophysiological confirmation of canonical movement-related changes 1 s before and 1 s after 40 repetitions of a cued contralateral arm movement, on-medications, aligned at the time of movement execution. Movement is associated with reduction in beta band (13–30 Hz) activity at both recording sites, with a concomitant increase in cortical broadband (50–200 Hz) (Crone et al., 1998).

We recorded neural data from a 57-year-old right-handed engineer and avid skier with a 10-year history of PD that began with reduced right arm swing. Preoperatively, he had motor fluctuations, rigidity, bilateral tremor (right>left) and gait disorder. Unified Parkinson’s Disease Rating Scale (UPDRS) scores were 34/9 off and on-medication, respectively. Lateralized motor scores during his off state were 10 for the left and 17 for the right body, showing that the left brain and right body were more affected. Recordings were performed using two bidirectional implanted pulse generators (IPGs) (Medtronic model Summit RC + S), each connected to an ipsilateral subthalamic cylindrical lead (Medtronic model 3389) and an ipsilateral subdural paddle-type lead (Medtronic model Resume II) over the sensorimotor cortex, approximately 25 mm from the midline (Figure 1A) (Gilron et al., 2021). Two-channel time series of bipolar local field potential recordings from the dorsal subthalamic nucleus and motor cortex, sampled at 500 Hz, were wirelessly transferred from each IPG to a tablet computer. The tablet computer was carried in a small backpack by the skier during all recordings. Successful data streaming was confirmed on the tablet computer before and after each skiing run, and recording sessions were kept under 10 min. Recordings were done in two different settings (i) in-clinic 10 days after surgery, on-medication, during a computer-controlled arm movement task consisting of a touchscreen-based center-out reaching task (Gilron et al., 2021). Briefly, each trial began with a ‘hold’ period of 5–7 s, during which the subject was instructed to rest their hand on a desk while maintaining gaze on a central target. The patient was then instructed to touch the target with his index finger, performing a continuous movement lasting approximately 1–2 s; and (ii) outdoors, 6 weeks after surgery, during downhill skiing and at rest on the chairlift, off-medication (4 h after early morning dose) and on-medication (30 min after habitual dose). During the ski runs, the patient made continuous wide turns, occasionally stopping for about 30 s between turns. Two skiing runs and one chairlift resting period were recorded for each medication state. Each skiing run lasted approximately 4–5 min, depending on the number and duration of stops per run, while each resting period on the chairlift lasted approximately 4 min.

All recordings were performed prior to initiation of chronic therapeutic stimulation. While skiing, video was aligned to neural activity by using a synchronization trigger that created a variable pulse width analog signal that was captured by both IPGs and recorded by the camera. Data were analyzed in 30 s segments, and segments containing movement artifacts were manually removed after visual inspection. We calculated power spectral density (PSD) and coherence using the multitaper method (1,000 ms windows with 500 ms overlap, 3 tapers and a time-half bandwidth product of 5). For the in-clinic movement task, 40 trials were aligned relative to the onset of movement and averaged. The averaged amplitude was normalized by the mean over the 1 s window prior to cue presentation. Data were z-scored by subtracting the average baseline amplitude and dividing by the baseline standard deviation, separately for each frequency. The postoperative localization of subthalamic electrodes were performed using the Lead-DBS toolbox and the integrated FieldTrip-SimBio pipeline in MATLAB (Horn and Kühn, 2015).

## 3 Results

**Figure 2:**
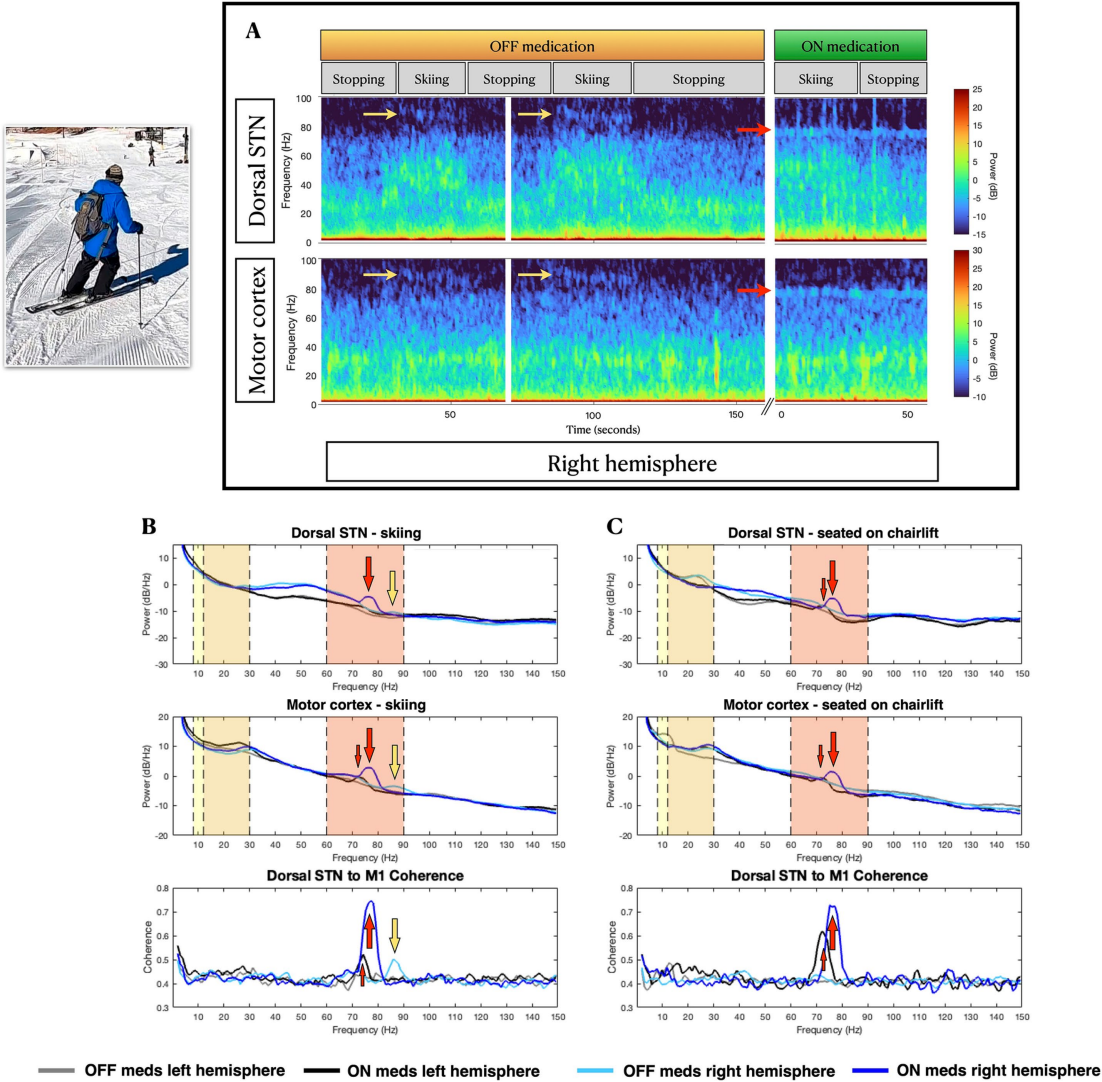
Skiing-related neural activity. **(A)**, Example spectrograms of right hemisphere subthalamic and cortical field potentials at stop-move transitions in the off-medications state (left), and during on-medications at rest (right). **(B,C)** Power spectral density of field potentials recorded from subthalamic nucleus (top row) and motor cortex (middle row), and subthalamic-frontal coherence (bottom row), during **(B)** skiing and **(C)** at rest on the chairlift, in off and on-medication states. In the right hemisphere, skiing induces narrowband gamma activity at 90 Hz (yellow arrows in **A** and **B**), while levodopa-induced narrowband gamma (large red arrows for right hemisphere, small red arrows for left hemisphere) occurs at a lower frequency (75 Hz). In the more severely affected left hemisphere, 75 Hz levodopa-induced gamma is less prominent than on the right, and the off-medication, skiing-induced 90 Hz narrowband gamma is absent. Vertical color bands delineate relevant frequency bands: yellow for alpha (8-12 Hz) frequencies, orange for beta (13–30 Hz) frequencies and red for high-gamma (60–90 Hz) frequencies.

In-clinic recordings (all performed on-medication) showed canonical movement-related cortical and subthalamic beta desynchronization and a cortical broadband gamma increase (Crone et al., 1998) (Figure 1B). In the subthalamic nuclei bilaterally, skiing had similar effects to the in-clinic task regarding beta desynchronization, but it additionally induced spectrally broad low-gamma activity at 30–60 Hz, the latter being more evident in the less affected (right) hemisphere (Supplementary Video 1 and Figures 2A,B). Conversely, in the motor cortex, high beta activity (20–30 Hz) remained prominent during ski turns, similar to stopping between ski turns, both on and off-medication, possibly because the participant generally maintained a steady outstretched arm posture throughout the ski run (Supplementary Video 1), while the in-clinic task required fluid distal arm movements.

During the skiing day, the on-medication state produced the expected reduction in STN beta activity (Figure 2C, top panel) and induction of finely-tuned gamma (FTG) activity at 75–80 Hz in STN and motor cortex, bilaterally (Figures 2B,C). Interestingly, skiing induced a distinctive FTG at 85 Hz (Supplementary Video 1 and Figures 2A,B), in both STN and cortex, only in the off-medication state, and only in the less affected (right) hemisphere. Corresponding increases in FTG fronto-subthalamic coherence were seen. This off-medication skiing-induced FTG differed in frequency from levodopa-induced FTG (Figure 2).

On the left hemisphere, prominent 10 Hz cortical activity was present during stopping, which may correspond to his resting state right arm tremor (Timmermann et al., 2003) (seen in the video while stopped). The lack of a left cortical beta peak during rest in the off-medication condition (Figure 2C) may be explained by the well-known phenomenon of tremor-related beta decrease (Qasim et al., 2016).

## 4 Discussion

With the use of sensing-enabled neurostimulators and real time wireless data transmission, it is now possible to collect multisite invasive brain data during almost any activity, and to synchronize these data to external monitors such as video. One limitation of this study is the potential changes in oscillatory dynamics during the weeks immediately after surgery, when the recordings were performed. However, while it is true that a microlesional effect can influence oscillatory activity in the STN, the literature suggests that this effect is mainly associated with beta frequencies (Peng et al., 2024). In this case, we did not find differences in beta-band dynamics between the two recording sessions. Additionally, the microlesional effect on this patient’s symptoms was mild and had mostly resolved by the time the in-clinic recordings were obtained. Although anecdotal in nature, the case provides interesting insights. First, complex self-cued movements during a highly learned sport may bring out different frequency bands compared to standard cued tasks or compared to normal spontaneous in-home activities, such as spectrally broadband low-gamma activity in the on and off-medication, off stimulation state. Second, the skiing-related FTG power and coherence observed were different in frequency compared to levodopa-induced FTG, suggesting a different mechanism of neural synchronization. And third, consistent differences between hemispheres in this subject with asymmetric motor signs, suggest that disease severity might play a role in the neural signatures of more complex motor tasks (Shreve et al., 2017). Identifying movement-related changes in oscillatory activity might also have a role in defining biomarkers that can work as a control signal for adaptive DBS. While 75 Hz levodopa-induced FTG might be a marker of an increased dopaminergic state and eventually linked to dyskinesias (Olaru et al., 2024), the 90 Hz movement-induced FTG might be a normal physiological phenomenon that occurs during some types of movement. These findings support the use of personalized algorithms of adaptive DBS, as recently suggested (Oehrn et al., 2024).

## Data Availability

The original contributions presented in the study are included in the article/Supplementary material, further inquiries can be directed to the corresponding author.

## Supplementary material

SUPPLEMENTARY VIDEO 1 Skiing-related neural activity. Example spectrograms of cortical and subcortical recordings during one run of downhill skiing in the off-medication state. Bilateral subthalamic **(A,C)** and motor cortex **(B,D)** spectrograms are synchronized with a video recording during skiing and stopping. Skiing is associated with a reduction in beta band activity (13–30 Hz) in both subthalamic nuclei, but not in the motor cortices. A tremor-related 10 Hz cortical activity in the left hemisphere is observed only during stopping. In the right hemisphere, skiing induces narrowband gamma activity at 90 Hz, but this activity is absent in the more severely affected left hemisphere.
